# Extraction of Essential Oils from *Lavandula × intermedia* ‘Margaret Roberts’ Using Steam Distillation, Hydrodistillation, and Cellulase-Assisted Hydrodistillation: Experimentation and Cost Analysis

**DOI:** 10.3390/plants11243479

**Published:** 2022-12-12

**Authors:** Jessie Wainer, Adrianne Thomas, Tania Chimhau, Kevin G. Harding

**Affiliations:** School of Chemical and Metallurgical Engineering, University of the Witwatersrand, Johannesburg 2000, South Africa

**Keywords:** energy consumption, hydrosol, lavender, scale-up, smell

## Abstract

Lavender oil is an important essential oil with many applications. The purpose of this study was to compare different methods of essential oil extraction to determine which method would be the most effective and profitable for commercial-scale production from *Lavandula × intermedia* (‘Margret Roberts’) flowers and leaves. The lavender from this variety flowers year-round, providing an extended production season compared to some other lavender varieties. Steam distillation, hydrodistillation, and cellulase-assisted hydrodistillation were used to extract oil. The average extraction times for steam distillation, hydrodistillation, and cellulase-assisted hydrodistillation were 57-, 51-, and 49 min, respectively, and the average energy consumption was 15.0-, 13.4-, and 30.8 kJ/g, respectively. Cellulase-assisted hydrodistillation produced the best quality oils, with a lower camphor content and a sweeter, more pleasant smell, while steam-distilled oils had the highest camphor content, as well as a more plant-like smell. Factors affecting scale-up (surface area of cut plants, equipment loading times, energy efficiencies, safety, mixing) have been discussed, while a basic cost analysis of theoretical large-scale processes showed that hydrodistillation and cellulase-assisted hydrodistillation would be the most and least profitable methods, respectively. Overall, hydrodistillation is recommended as the best method for commercial lavender oil production.

## 1. Introduction

Essential oils are defined as the volatile components of a plant which give the plant its distinctive smell [1]. They are hydrophobic liquids containing saturated and unsaturated hydrocarbons and oxygenated compounds, including alcohols, esters, ketones, aldehydes, and terpenes [2].

Lavender oil is among the most widely used essential oils, as well as one of the most profitable specialty crops [3], with applications including aromatherapy, food flavoring, cosmetics, and detergents, with the benefits listed as antiseptic and anti-inflammatory [4].

The species of lavender most used for oil extraction are *Lavandula angustifolia* (English lavender) and *Lavandula × intermedia* (a hybrid of English lavender and Portuguese lavender). *Lavandula × intermedia* typically provides a higher yield of oil, but the oil is of lower quality and has a higher camphor content than oil from *Lavandula angustifolia* [5].

There is promising potential for the South African essential oil industry to grow and thrive [6]. Research on the oil extraction methods is therefore important for South African essential oil producers to select a method which optimizes oil quality and profitability.

The two conventional methods of essential oil extraction are hydrodistillation and steam distillation. Both methods use heat to vaporize the oil, allowing it to escape the plant. The separation of water and oil is made possible by the difference in the density of the two components. In hydrodistillation, the lavender is placed in boiling water, whereas, in steam distillation, only the steam passes through the lavender. Both methods are energy-intensive and time-consuming, and there is an abundance of current research investigating how to improve and adapt these methods to produce more oil of better quality, while reducing energy consumption and extraction time [7].

One method suggested to improve distillation is to treat the plant material with an enzyme before extraction. The enzyme is expected to break down the walls of the plant cells, making the oil easier to extract [8]. In the extraction of oil from garlic, thyme, rosemary, and spices, enzymatic pre-treatment has been found to significantly increase both the overall yield of oil and the yield of major desirable components in the oil [8,9,10].

Steam distillation is the extraction method currently used for most commercial essential oil production. Research suggests that other methods may have many advantages over steam distillation, such as shorter extraction time, lower energy consumption, and better oil quality [7]. Before these methods can be used for commercial processes, they must be assessed on a small laboratory scale to determine their feasibility for large-scale oil production. The results from small-scale experiments can be used to predict which method would be best suited to commercial-scale production.

This study aimed to develop an efficient, effective, and profitable lavender oil extraction method which can be used for commercial-scale production. Three methods of essential oil extraction were investigated—steam distillation, hydrodistillation, and cellulase-assisted hydrodistillation—to determine the method which would be best suited to a scale-up. The extraction time, oil quality, and energy consumption were compared. A theoretical cost analysis of a large-scale process is also presented. The lavender used for the extraction experiments was Margaret Roberts lavender, classified as *Lavandula × intermedia* ‘Margaret Roberts’, or *Lavandula × heterophylla*, grown at the Margaret Roberts Herbal Centre in Hartbeespoort, South Africa, and harvested at the beginning of spring.

## 2. Results and Discussion

### 2.1. Extraction Time

In this experiment, the extraction time refers to the total time for distillation, starting from room temperature, increasing the temperature up to the time when the separator reached 200 mL, i.e., it does not include the pre-treatment time. The average extraction times for steam distillation, hydrodistillation, and cellulase-assisted hydrodistillation were 57-, 51-, and 49 min, respectively. Cellulase-assisted hydrodistillation had the shortest extraction time. In this method, the plant mixture was already at 40 °C following pre-treatment, which explains why it heats up and boils more quickly. Shorter extraction times are more desirable to reduce costs; however, it must be noted that the total process time for cellulase-assisted hydrodistillation was significantly longer than that of the other methods because of the 45 min pre-treatment stage. This is discussed further in the section on energy consumption (Section 2.4).

It was expected that steam distillation would have the longest extraction time, because this method’s set-up required more glassware than the hydrodistillation methods, and the steam must travel farther before reaching the condenser. Furthermore, unlike hydrodistillation, steam distillation requires the steam to travel through tightly packed plant matter. This factor could be mitigated, to some extent, by placing packing in the section with the plant matter to allow an easier flow of steam through the lavender. The plant matter should, however, not be too spread out; otherwise, the steam would flow around instead of through the plant, and would not pick up oil.

To decrease extraction times, the heating mantle could be set to a slightly higher setting. However, care would need to be taken to ensure there were no adverse effects of high temperatures, particularly in the enzymatic system.

It should be noted that extraction time for a specific volume of oil, and not total distillation volume, may be a more suitable measurement, in some cases, but this was not possible to measure in the setup provided here.

### 2.2. Oil Quality

**Figure 1:**
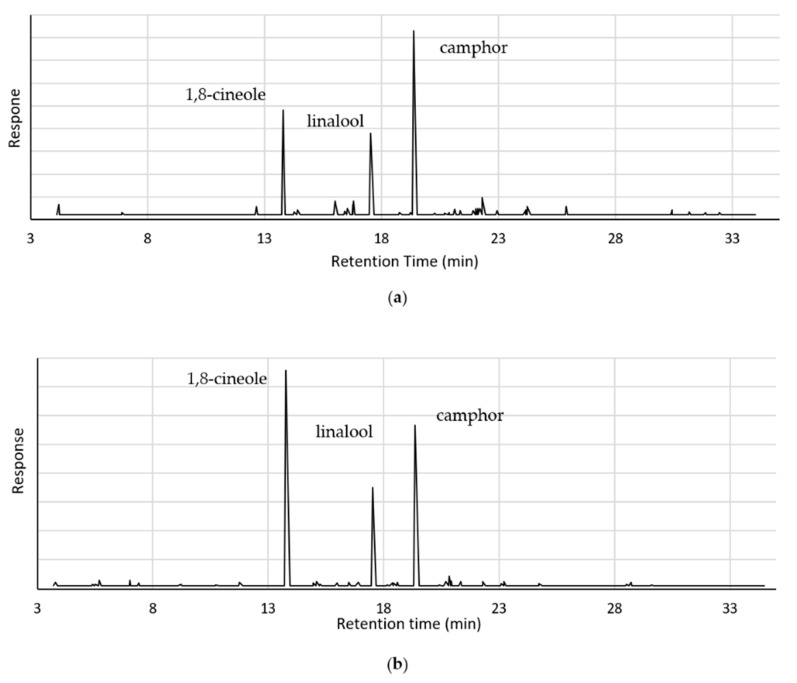

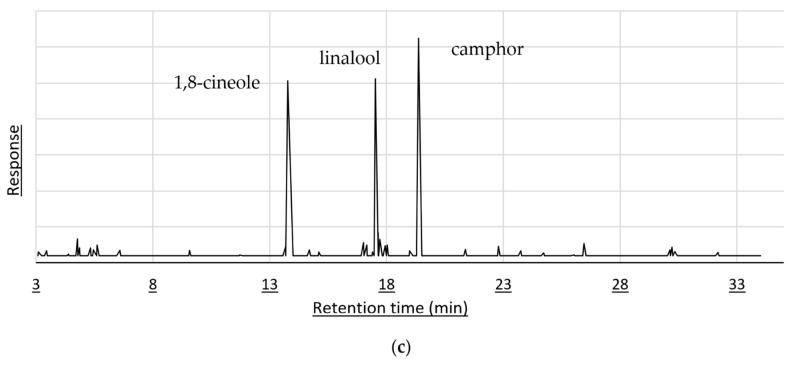
GC-MS chromatograms for lavender oil samples extracted using (**a**) steam distillation, (**b**) hydrodistillation, and (**c**) cellulase-assisted hydrodistillation.

The oil quality was determined by detecting the major compounds in the oil, and their concentrations. The concentrations of the various components in the oil samples were compared by comparing the relative areas of the peaks in the GC-MS chromatograms (Figure 1). The oil samples resulted in chromatograms that can easily be interpreted, as they have flat baselines and sharp, defined peaks. GC-MS analysis identified up to 35 components in each oil sample, but most had peak areas lower than 5% of the total peak area. Most chromatograms showed three significant peaks at retention times of about 13.8-, 17.5-, and 19.4 min.

The components of interest are those commonly reported to dominate *Lavandula × intermedia* and *Lavandula × heterophylla*, including linalool, linalyl acetate, 1,8-cineole (eucalyptol), and camphor, each usually present at a concentration of not more than 40% [11,12,13].

The concentrations of the main components present in the oil samples (those with peak areas above 5%) are shown (Table 1). All the oil samples contained some amount of 1,8-cineole and camphor. These two components correspond with the first and third peaks on the chromatograms. They are commonly reported as the main components in the analysis of lavender oil [11,14,15,16,17].

Linalool was also expected to be one of the main components of the oils, but was only present in five out of nine oil samples, and three contained terpinolene instead. Terpinolene has also been identified as one of the main compounds in the leaves and flowers of many species of lavender [18], but it is usually reported in low quantities (less than 2%) in lavender oil [19,20,21,22]. It has, however, been reported at a concentration of up to 22.22% in the Indian species *Lavandula gibsoni* [23].

There are three possible explanations for the unusually high terpinolene content in three of the oil samples. The most straightforward reason is that these oil samples actually do contain more terpinolene. Terpinolene is characterized by high antimicrobial and antifungal activities [24]; therefore, the high terpinolene content shows that the oil would be a suitable ingredient in soaps and detergents. Terpinolene is also effective as a sedative [25], which would make the oil effective in aromatherapy. However, this would not explain why three samples contained terpinolene, and the other five contained linalool.

The retention time reported for linalool was between 17.543 to 17.554 min, while the retention time for terpinolene was reported between 17.531 and 17.541 min. These very similar values suggest that the linalool peak on the chromatogram could have been erroneously interpreted as terpinolene. Taking into account that all of the oil samples are from the same flowers, that high linalool content is expected, but not high terpinolene content, and that none of the nine samples had both a high linalool content and a high terpinolene content, it would be reasonable to assume that the values reported for terpinolene were actually linalool.

Another explanation for the high terpinolene content is that there was originally linalool present in the samples, but it converted to terpinolene while in storage. Linalool can be chemically rearranged into a carbocation, which can then convert to terpinolene, which is more stable [26]. Some of the samples were in storage for up to three weeks before being analyzed, so the linalool was possibly converted to terpinolene.

It is, therefore, justifiable to interpret terpinolene concentrations as linalool concentrations. This interpretation means that all the samples contain 1,8-cineole, linalool, and camphor as their main constituents.

The higher camphor content and lower linalool concentration in all the oil samples in this study indicate that oil from Margaret Roberts lavender would be preferred for soaps, detergents, aromatherapy, and phytotherapy, and would not be as extensively used for perfume and cosmetics [16,18].

The datasets have high standard deviations. This reflects that although the experiments were carried out in triplicate, there is a low level of consistency in the GC-MS analysis results from different oil samples obtained from the same extraction method. Future experimentation should carefully monitor the temperature of the water, the time between harvesting and experimentation, and the time between pre-treatment and extraction; these are factors which may affect the consistency of the oil quality.

The standard deviation was smaller for the concentration of linalool than for the concentration of 1,8-cineole and camphor, showing that the concentration of linalool was a more consistent and predictable value. The concentrations of 1,8-cineole and camphor were more sensitive to changes in extraction conditions.

Hydrodistillation and cellulase-assisted hydrodistillation exhibited larger standard deviations than did steam distillation. Steam distillation produced more consistent results and was not as sensitive to small changes in experimental parameters.

Oils obtained from conventional hydrodistillation and cellulase-assisted hydrodistillation contained more 1,8-cineole and linalool than oils from steam distillation, while oil obtained from steam distillation contained more camphor. Camphor has a penetrating, minty odor [16], and oils with a high camphor content are, therefore, expected to have a strong smell and to be associated with a ‘cool’ odor.

When comparing hydrodistillation with cellulase-assisted hydrodistillation, the results suggested that the enzymatic pre-treatment step results in the oil containing less camphor than if there was no pre-treatment step. Cellulase-assisted hydrodistillation resulted in oils with the lowest ratio of camphor to linalool, indicating that these oils were of the best quality and could be used in higher-value products [18].

### 2.3. Oil Smell

To compare the smell of oils obtained using different extraction methods, three metrics were used: pleasantness, strength, and flavor (flavor is defined here as the characteristic words used to describe the oil, such as floral, sweet, and sour).

Oils obtained using cellulase-assisted hydrodistillation were rated as the most pleasant-smelling oils, with an average score of 4 out of 5, and oils obtained from steam distillation and hydrodistillation were rated equally, at an average of 3 out of 5. This suggested that the pleasant smell of the cellulase-assisted hydrodistillation oils could be attributed to the enzymatic pre-treatment, since this pre-treatment step is absent in the other two methods.

**Figure 2:**
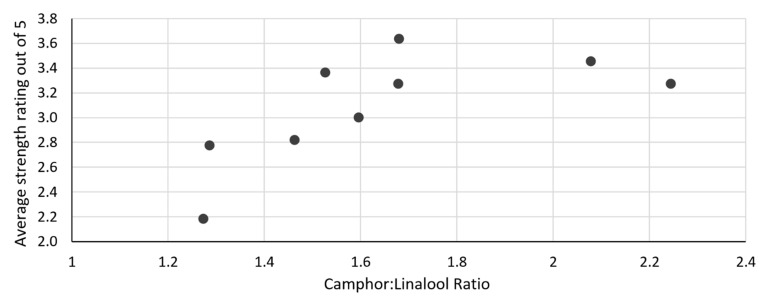
Smell strength of lavender oil samples versus their camphor-to-linalool ratios.

The lower camphor-to-linalool ratio present in the cellulase-assisted hydrodistillation oils was associated with its better quality and more pleasant smell compared to oils obtained from the other methods. However, cellulase-assisted hydrodistillation oils were also reported to have the weakest smell, reflecting the downside of the low camphor-to-linalool ratio. Figure 2 shows that oils with a higher camphor-to-linalool ratio generally had a stronger oil smell, but not if the ratio was increased above 2:1. The graph suggests that to obtain an oil with a strength rated 3 or above, the camphor-to-linalool ratio must be at least 1.5:1.

Oils with a weaker smell could either be used in applications where a more subtle smell is desirable or could be dried using anhydrous sodium sulfate. Drying removes the excess water and concentrates the oil, resulting in a more powerful smell. This drying step would likely not be necessary for steam-distilled oils, as these oils were rated as the strongest-smelling oils. The high strength of the smell of the steam distillation oils correlated with its higher camphor content, as discussed in the section on oil quality (Section 2.2).

The sensory analysis panelists associated all the oil samples with a floral, lavender flavor. This reflects that all the methods investigated in this study produced distinctive lavender oil, showing that Margaret Roberts lavender has good potential for lavender oil production, regardless of the extraction method. Six oil samples were also reported to have a ‘cool’ or refreshing flavor, suggesting that they would be suitable for use in detergents.

Oils from steam distillation were found to have a plant-like flavor. These oils could be used for aromatherapy or detergents. However, to be used for perfumery and cosmetics, the fragrance would have to be enhanced by adding sweeter-smelling compounds to create a more delicate, light smell.

Cellulase-assisted hydrodistillation produced oils which were consistently reported to have a sweet smell, more suitable for perfumes than oils obtained from the other methods. This suggests that a lower camphor content correlates with a sweeter smell.

### 2.4. Energy Consumption

The energy consumed by the different extraction methods was compared by estimating the energy used by the heater and mixer during experimentation. For a large-scale process, other factors, such as the machinery used to harvest and cut the lavender, would have to consider; however, these values are constant in all methods, so they were not included in this comparison. It is assumed that the heater operates at 70% of its maximum power rating, and the mixer operates at its maximum power rating.

**Figure 3:**
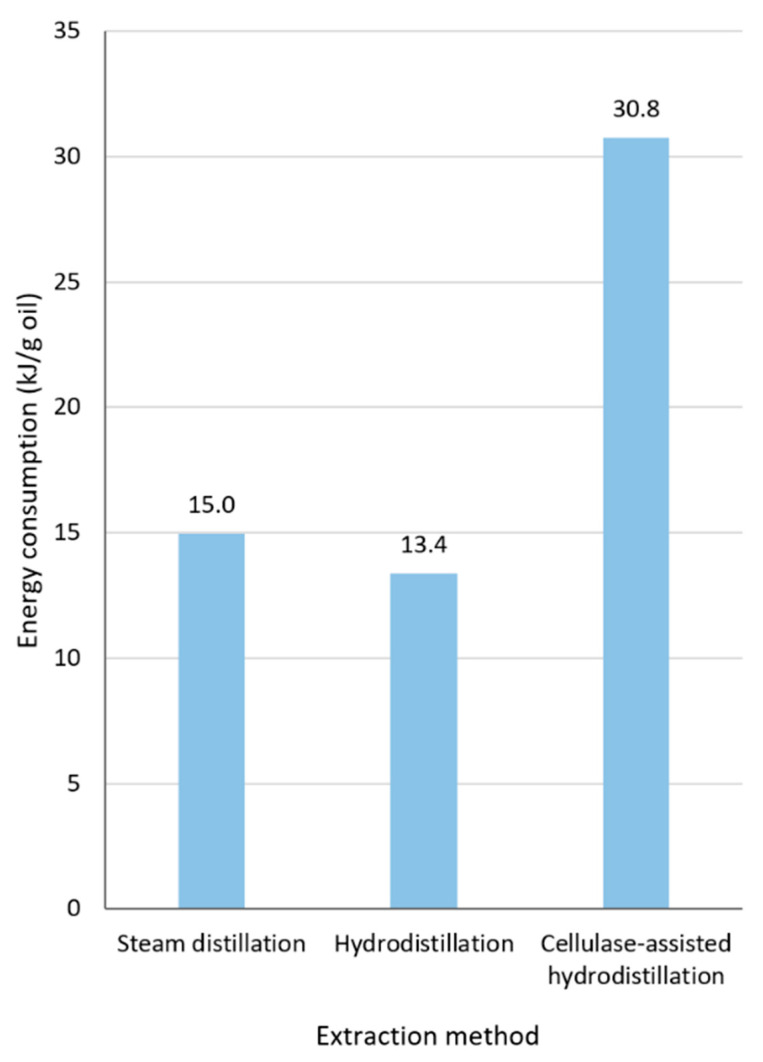
Energy consumption of three different lavender oil extraction methods.

The average energy consumption for steam distillation, hydrodistillation, and cellulase-assisted hydrodistillation are compared in Figure 3. As expected, steam distillation and hydrodistillation, which both only use the heater and not the mixer, exhibited similar energy consumption, whereas cellulase-assisted hydrodistillation showed significantly higher energy consumption. Hydrodistillation required slightly less energy than steam distillation because of its shorter extraction time. The enzymatic pre-treatment stage resulted in cellulase-assisted hydrodistillation having an energy consumption more than double that of conventional hydrodistillation.

## 3. Scale-Up

### 3.1. Factors Affecting Scale-Up

It cannot be assumed that oil extraction processes would scale up linearly from a laboratory scale to a commercial scale. Changing the sample size and equipment size changes the dynamics of the system. This section discusses important considerations for a theoretical scaled-up process using 1800 g of lavender plant material.

The large amount of plant material means that it could not easily be cut into very small pieces, as it was in the small-scale experiments, as this would take an unreasonable amount of time, if done manually, and add significant cost, if done mechanically. This means the surface area of the plant in contact with water or steam would be lower, thereby decreasing the mass transfer of oil from the plant. A large-scale process could, therefore, be expected to take longer to produce the same amount of oil, and the differences in extraction times between the three methods may be more pronounced on a larger scale.

During small-scale experiments, it was observed that loading the plant material into the glassware was a very time-consuming step, because of the small size of the flask opening. For a scaled-up process, there is a far larger amount of plant material, and the glassware for the plant material must, therefore, be designed with a large opening, or a removable lid, to prevent the loading of the plant material into the glassware from becoming too time-consuming.

The larger surface area of large-scale extraction glassware means that heat loss is expected to be high. The energy consumption, therefore, cannot be linearly scaled up. If the heating mantle could be designed to cover most of the glassware, this would minimize heat loss, and the efficiency would be high. It is assumed that energy consumption could be scaled up using an efficiency of 80%, i.e., 20% of the supplied energy would be lost.

For a large-scale steam distillation process, there is another consideration regarding energy consumption. In the small-scale set-up, the plant flask was not externally heated, but was kept hot by the inflow of steam. In a large-scale process, more heat would possibly be lost from this flask, and the incoming steam may condense before reaching the condenser. Therefore, a large-scale steam distillation process should incorporate a heating coil around the plant flask to maintain this section at a high temperature. The heating coil is estimated to increase the energy consumption of large-scale steam distillation by 30%.

Safety is an important consideration for scale-up. Far more water (at least 20 L vs. the original 1 L) would have to be boiled in a large-scale process than in the small-scale experiments, creating far more steam. Safety controls and careful monitoring would be necessary to mitigate the risks of the steam escaping or pressure building up in the system and causing glassware to shatter. To mitigate this problem, stainless steel equipment could be used instead of glass in the large-scale set-up. 

The enzymatic pre-treatment step would likely be more complex on a large scale. In small-scale experiments, the plant material had to be regularly mixed manually to avoid clumping on the surface of the water. In the scaled-up process, since there is more plant material, it would require more agitation to maintain thorough mixing for the 45 min pre-treatment time.

The quality and smell of oils extracted in large-scale extraction are not expected to change drastically from those obtained at the small-scale. It would, however, be easier to dry the oil samples to a suitable level because there would be a significantly larger amount of oil. Drying the oil would make it possible to accurately measure the oil yield and would result in the oils having a stronger smell.

### 3.2. Scale-Up Cost Analysis

A basic cost analysis was used both to determine if a large-scale process would be profitable and to compare the profitability of the processes using the three different methods investigated in this study. This cost analysis assessed a large-scale process which extracts oil from 1800 g of lavender.

The yield of lavender oil ranges from 0.5% to 6.8% (by mass), and most studies report a yield between 4% and 6% [7,15,27,28]. Assuming a yield of 5%, extraction using 1800 g of plant matter would result in 90 g of oil. The density of this type of lavender oil is estimated to be 0.895 g/mL [29]; therefore, the yield would be 100.6 mL of oil. Lavender oil is generally sold in 10 mL vials, so 10 vials per batch could be sold from a large-scale process.

It is assumed that the oil from cellulase-assisted hydrodistillation extraction would be the most expensive due to its lower camphor content, and conversely, the oil from steam distillation would be the least expensive, as it has the highest camphor content. Based on retail essential oil prices, one 10 mL vial of oil from steam distillation, hydrodistillation, and cellulase-assisted hydrodistillation would be sold for an estimated USD 5.40, USD 6.00, and USD 6.60, respectively.

The hydrosol obtained in this study had a very pleasant fragrance, and it is therefore assumed that this could be sold as a room spray. The small-scale experiments with 80 g of plant matter yielded one bottle of hydrosol. A large-scale process would yield 22 bottles of hydrosol, with an assumed sale price of USD 5 each.

The total cost of oil extraction includes the costs of flowers, cellulase, electricity, and packaging. Margaret Roberts lavender costs ZAR 80 (South African Rand, the equivalent of USD 5.33) for a bunch of flowers weighing approximately 110 g. Cellulase from Sigma-Aldrich costs roughly USD 113.80 per 50 mL. The average cost of electricity in South Africa is ZAR 1.07/kWh (USD 0.07/kWh) [30]. Electricity costs are dependent upon energy consumption, which was calculated using the efficiencies discussed in the section on factors affecting scale-up (Section 3.1). Amber glass vials for the oil and plastic spray bottles for the hydrosol are estimated to cost USD 1 each.

Because of the high price of the cellulase enzyme, cellulase-assisted hydrodistillation is not expected to be profitable (Table 2). Hydrodistillation is the most profitable process, yielding a profit of USD 50, or USD 28 per kilogram of lavender plant material.

## 4. Materials and Methods

### 4.1. The Plant Material

The lavender was briefly stored in vases of water immediately after harvesting, and then shortly before experimentation, it was air dried in the shade for a few days, cut into pieces of an average size of 1 cm, and weighed. The spike (flowers), and approximately 30 cm of the stem, were used for extraction.

### 4.2. Extraction Methods

#### 4.2.1. Steam Distillation Procedure

**Figure 4:**
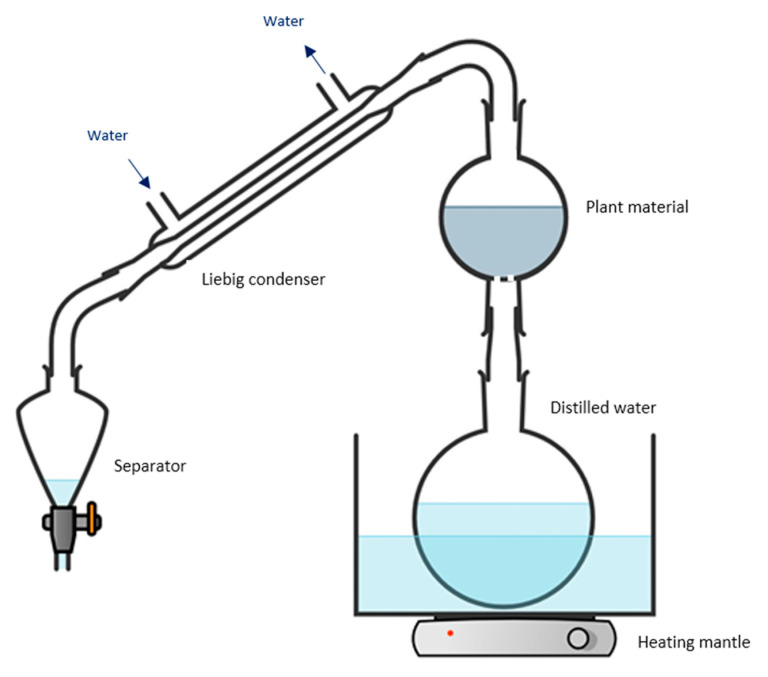
Schematic diagram of the steam distillation set-up. (Figure created by the authors: https://chemix.org/ (accessed on 10 October 2020)).

A two-neck round-bottomed glass flask with one opening on the top and the other on the bottom was used for the experiments. Mesh was placed to cover the bottom opening, and 80 g of cut lavender was loaded through the top opening. Distilled water was boiled, and the steam was directed into the bottom opening of the lavender flask. The oil-rich steam exiting the top of the flask was directed into a Liebig condenser, and then into a separator funnel (Figure 4). The extraction ran until 200 mL were deposited into the separator, and the time was recorded. After extraction, the separator contained oil floating on hydrosol (aromatic water). The hydrosol was drained, and the oil was stored.

#### 4.2.2. Hydrodistillation Procedure

**Figure 5:**
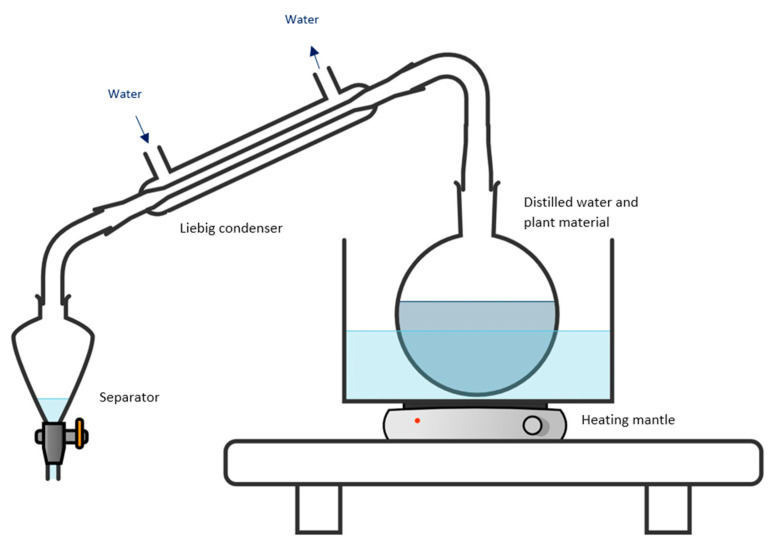
Schematic diagram of the hydrodistillation set-up. For cellulase-assisted hydrodistillation, the cellulase is in the flask with the distilled water and plant material. (Figure created by the authors: https://chemix.org/ (accessed on 10 October 2020)).

The same amount (80 g) of plant material was used for hydrodistillation. The plant material and 1 L of water were placed in a round-bottom flask and heated, and the steam was directed into a Liebig condenser, and then into separator funnel (Figure 5). When the separator level reached 200 mL of hydrosol, the oil was separated, collected, and stored.

#### 4.2.3. Cellulase-Assisted Hydrodistillation Procedure

The plant material (80 g) was pre-treated by mixing it with 1 L distilled water and 5 mL of cellulase for 45 min using a magnetic stirrer, and the mixture was maintained between 40 °C and 45 °C. The mixture was then moved to the hydrodistillation set-up for extraction.

### 4.3. Materials and Equipment

The enzyme used for pre-treatment was Celluclast 1.5 L cellulase (Novozyme, Bagsværd, Denmark), which has an activity of 80 FPU/mL (filter paper units—as from the supplier). The cellulase was stored in an autoclaved Schott bottle at 0 °C until it was used.

The heating mantle used for the experiments had a power rating of 500 W and was used at 70% of its maximum setting, and a magnetic stirrer was used at its maximum setting of 530 W.

After extraction, the oil was stored at room temperature in sealed glass vials covered in aluminum foil. All experiments were carried out in triplicate.

### 4.4. Analysis

#### 4.4.1. Chemical Composition

To identify the components present in the oil, gas chromatography with mass spectrometry (GC–MS) was used. The GCMS-QP2010 Ultra (Shimadzu, Kyoto, Japan) was used for this analysis. Each oil sample was run for 35 min. Hexane was used as a solvent, and helium was used a carrier gas at 200 kPa. The conditions of the GC portion were: an injection temperature of 210 °C, oven temperature of 40 °C, ‘split’ injection mode, a total flow of 50.0 mL/min, and column flow of 1.78 mL/min. The mass-to-charge size targeted for MS was 45 to 500 m/z. The MS conditions were: an ion source temperature of 210 °C and an interface temperature of 250 °C. The components were identified by comparing the GC-MS data with the NIST14 and Wiley9 libraries.

#### 4.4.2. Sensory Analysis

Twelve panelists smelled the oil samples and rated the pleasantness of the odors on a scale of 1–5, where 1 indicated an unpleasant smell, and 5 indicated a very pleasant smell. The strength of the odors was also rated on a scale of 1–5, where 1 indicated a very faint smell, and 5 indicated a very strong smell. The panelists described the smells in their own words, and also picked the dominant smells from a set list of words (plant-like, floral, sweet, cool, sour, woody, fatty, spicy, and herbaceous).

## 5. Conclusions

This study compared steam distillation, hydrodistillation, and cellulase-assisted hydrodistillation for the extraction of lavender oil. Hydrodistillation is the overall best method for lavender oil extraction. It exhibits a reasonable extraction time, oil quality, and oil smell, and has the lowest energy consumption of the three methods. It is also the simplest method, with a basic set-up that can easily be scaled up. Most significantly, it is predicted to be the most profitable method on a large scale and is therefore recommended as the best method for commercial-scale lavender oil production.

The excellent quality and smell of oils obtained from cellulase-assisted hydrodistillation suggest that further research into this method would be worthwhile. If a different, cheaper enzyme could be used with the same effect, it may make this an economically competitive method.

## Figures and Tables

**Table 1:** Composition of oil samples extracted using three different methods.

Extraction Method	Steam Distillation			Hydrodistillation			Cellulase-Assisted Hydrodistillation		
**Sample #**	**1**	**2**	3 *	**4**	**5**	**6**	**7**	**8**	**9**
**1,8-cineole (wt%)**	18.55	14.18	-	13.96	15.6	27.91	26.82	17.04	13.48
**Linalool (wt%)**	-	13.67	-	16.96	21.89	-	20.18	-	15.35
**Terpinolene (wt%)**	18.56	-	-	-	-	14.86	-	17.06	-
**Camphor (wt%)**	28.34	30.68	-	24.82	36.74	24.97	32.21	21.73	19.75

* Analytical equipment gave obviously incorrect results and could not be repeated (time and sample limitations).

**Table 2:** Comparison of the income and expenditure of the lavender oil extraction processes using three different extraction methods (costs in USD).

	Steam distillation	Hydrodistillation	Cellulase-Assisted Hydrodistillation
**Expenditure**			
**Flowers**	87	87	87
**Cellulase**	0	0	256
**Electricity**	0.87	0.60	1.37
**Amber vials**	10	10	10
**Hydrosol bottles**	22	22	22
**Income**			
**Oil**	54	60	66
**Hydrosol**	110	110	110
**Profit/Loss**			
**Profit/Loss**	44	50	−201

## Data Availability

Data is contained within the article or on request from the authors.
